# Reproducibility of Thermography for Measuring Skin Temperature of Upper Limbs in Breast Cancer Survivors

**DOI:** 10.3390/biomedicines12112465

**Published:** 2024-10-27

**Authors:** Vanessa Maria da Silva Alves Gomes, Naiany Tenório, Ana Rafaela Cardozo da Silva, Laura Raynelle Patriota Oliveira, Ana Claúdia Souza da Silva, Juliana Netto Maia, Marcos Leal Brioschi, Diego Dantas

**Affiliations:** 1 Departamento de Fisioterapia, Centro de Ciências da Saúde, Universidade Federal de Pernambuco, Recife 50670-901, Brazil; vanessaalvesfta@gmail.com (V.M.d.S.A.G.); n.tenorio.j@gmail.com (N.T.); rafaela.cardozo@ufpe.br (A.R.C.d.S.); laurapatriotaa@gmail.com (L.R.P.O.); claudia.souzasilva@ufpe.br (A.C.S.d.S.); juliana.netto@ufpe.br (J.N.M.); 2 Medical Thermology and Thermography Specialization, Hospital das Clínicas da Faculdade de Medicina da Universidade de São Paulo, São Paulo 05508-220, Brazil; marcosbrioschi@gmail.com

**Keywords:** thermography, lymphedema, breast cancer survivors, skin temperature, reproducibility

## Abstract

**Background/Objectives:** Breast cancer-related lymphedema (BCRL) is a chronic condition that has early diagnosis as a critical component for proper treatment. Thermography, a non-invasive imaging method, is considered a promising complementary tool for the diagnosis and monitoring of BCRL, especially in subclinical stages. The present study aimed to evaluate the intra- and inter-examiner reproducibility of thermography for measuring the skin temperature of the upper limbs (UL) of women with and without BCRL. **Methods**: This study, conducted with women who underwent a unilateral mastectomy, assessed BCRL using indirect volumetry. Maximum, minimum, and mean skin temperatures were measured in five regions of interest (ROI) of each UL (C1, C2, C3, C4, and Cup) in four different postures. Reproducibility measures were assessed using an intraclass correlation coefficient, 95% confidence interval, and coefficient of variation. **Results**: The sample comprised 30 women; 14 were diagnosed with BCRL. A total of 120 thermograms were recorded in different postures, and 3600 ROI were analyzed in the UL with and without BRCL. The intraclass correlation coefficient of the analyses indicated intra- and inter-examiner reproducibility from good to excellent (0.82 to 1.00) for all skin temperatures evaluated (maximum, minimum, and mean). The coefficient of variation for all measures was below 10%, indicating low variability. **Conclusions**: Our findings demonstrate that thermography shows good-to-excellent reproducibility across multiple postures and regions of interest, reinforcing its potential as a non-invasive and reliable method for assessing lymphedema in breast cancer survivors. This study establishes a foundation for incorporating thermography into clinical practice for early BCRL detection, particularly in subclinical stages, thus improving patient management and outcomes.

## 1. Introduction

Survival rates of breast cancer, the most common among women worldwide, increased due to advances in treatment, including chemotherapy, radiotherapy, hormone therapy, and surgery. In this sense, concerns about long-term complications that may impair quality of life, such as breast cancer-related lymphedema (BCRL), have also risen [1]. BCRL is the accumulation of fluid in interstitial tissues, with the presence of fat and tissue fibrosis. Its manifestation may occur late, emphasizing the importance of monitoring and early diagnosis for optimizing long-term prognosis and facilitating effective treatments [2].

The diagnosis of BCRL relies on a detailed clinical evaluation encompassing medical history, physical examination, and imaging. Although effective, conventional methods like bioimpedance spectroscopy and lymphoscintigraphy are limited due to the high cost and accessibility [3]. Despite being indirect, perimetry is the most used method in clinical practice due to its accessibility, even with the inability to detect BCRL in subclinical stages (i.e., no changes in the limb volume) [4]. In this context, alternative diagnostic methods are crucial for a detailed analysis of tissue alterations in BCRL, and thermography emerges as a viable and promising complementary exam for the diagnosis of BCRL. This non-invasive technique assesses the infrared energy emission of the skin, reflecting metabolic and circulatory changes characteristic in BCRL [5,6].

Recent studies have highlighted cutaneous thermography as a promising tool for managing BCRL [5,6]. In addition to being non-invasive, thermography detects real-time thermal variations in the skin, indicating changes in circulation and fluid distribution, even in the absence of visible changes in limb volume or size. Thermography has also proven to be a non-invasive tool for assessing lower limb lymphedema, demonstrating its potential for point-of-care diagnosis in clinical settings [7]. Despite the positive emphasis on the broad clinical applicability of thermography for assisting in the diagnosis and monitoring of various health conditions, including BCRL [8], it is essential to investigate the reproducibility of thermography to ensure its practical utility and to establish the necessary requirements for reliable image acquisition.

Despite its potential, there is still a lack of standardized protocols for using thermography in the assessment of upper limbs in breast cancer survivors. The reproducibility of thermographic measurements in this context remains underexplored, especially in relation to the standardization of patient positioning and device calibration. External factors such as an ambient temperature and humidity can also influence the accuracy of the results, further highlighting the need for controlled environments during assessments. Addressing these gaps is crucial to ensure the reliability of thermography in clinical practice.

Hence, this study aimed to assess the intra- and inter-examiner reproducibility of thermography in measuring the skin temperature of the upper limbs (UL) of women with and without BCRL. By investigating temperature across five regions of interest and four distinct postures, this study seeks to determine which posture(s) and region(s) provide the most consistent and reliable thermographic assessments, ultimately offering recommendations for clinical use.

## 2. Materials and Methods

### 2.1. Study Design and Sample

This cross-sectional and prospective study was conducted in the oncology physiotherapy outpatient of the Department of Physiotherapy at the Universidade Federal de Pernambuco. This study was approved by the research ethics committee (no. 57624121.00.000.5208), and all participants signed the informed consent form. This study adhered to the principles of the Helsinki Declaration.

This study included 30 survivors of breast cancer aged between 40 and 70 years with a history of unilateral mastectomy. Exclusion criteria were history of bilateral breast cancer, primary lymphedema, edema from other causes (rheumatologic, renal, neurological, orthopedic, or previous vascular disease), pre-existing skin conditions affecting thermoregulation (e.g., erysipelas, intertrigo, or ulcers), and ongoing chemotherapy or radiotherapy treatment.

### 2.2. Recruitment and Screening

Participants were recruited from clinics, hospitals, and digital media promotions. The screening was performed at Universidade Federal de Pernambuco using a form containing the inclusion and exclusion criteria. A dermatologist then reviewed each participant, verifying eligibility based on predefined criteria. Eligible women signed the informed consent form following resolution 466/12 of the Brazilian National Health Council.

### 2.3. Data Collection

Examiners were trained for all planned measurements, including medical history, physical examination, acquisition, and analysis of thermographic images. Participants completed a form with the following clinical data: age, height, weight, dominant side, affected UL, date of cancer diagnosis, date and type of surgery, and other treatments.

**Figure 1:**
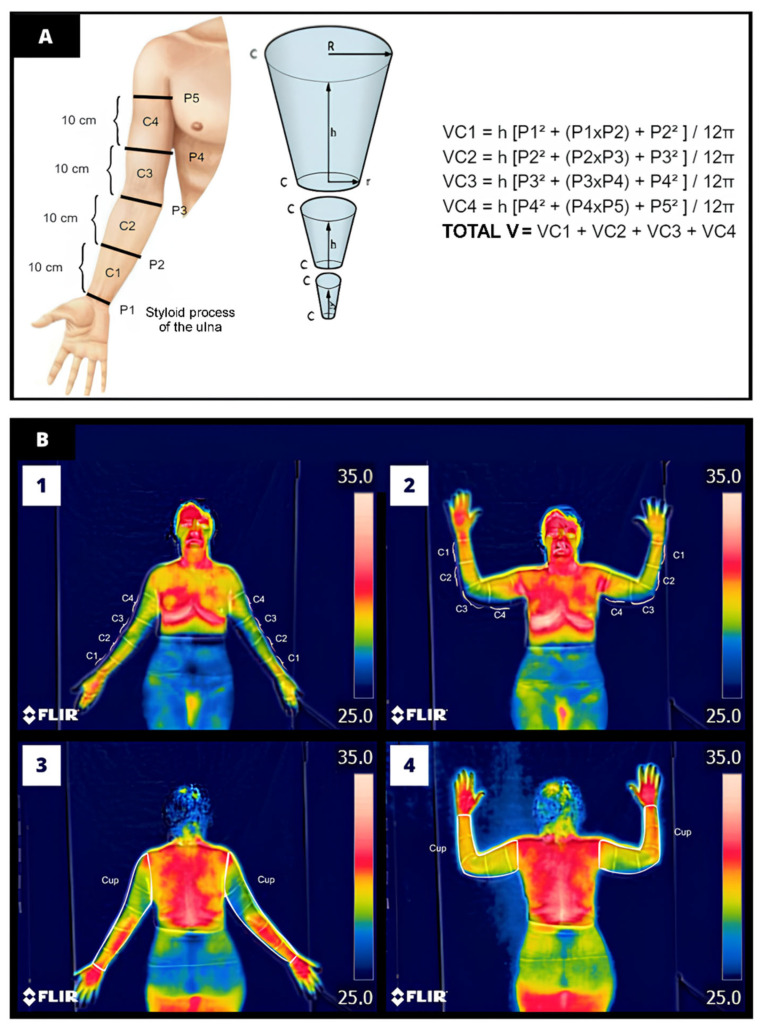
Representative illustration of (**A**) indirect volumetry technique for diagnosing upper limb lymphedema, VC is the final volume of the limb segment (VC1—volume of compartment 1 that is between the perimetries (1 and 2); P: perimetry; h: distance between the perimetries (10 cm); π = 3.14159. (**B**) Manual delineation of the region of interest (ROI) performed by the examiners. Figures B1 and B2 illustrate the delineation of ROI C1 to C4 in the anterior anatomical and anterior arm abduction postures, respectively. Panels B3 and B4 exemplify the delineation of the ROI Cup encompassing the entire upper limb in the posterior anatomical and posterior arm abduction postures, respectively.

The standard assessment of BCRL followed the International Society of Lymphology guidelines, which consisted of indirect volumetry via cone volume calculation (Figure 1) [9]. This method offers good diagnostic accuracy and reproducibility by comparing the volume of the affected and unaffected limbs [10,11]. Circumferences were measured in standing position, one UL at a time. Participants were instructed to raise the shoulder to a 90-degree angle in abduction and external rotation, with the elbow extended, wrist in ulnar deviation, and open hand against the wall. In this position, five equidistant measures of 10 cm were marked with a dermatographic pencil. The first marking (P1) was referenced by the styloid process of the ulna, while subsequent measures (P2, P3, P4, and P5) were delineated until reaching 40 cm from P1 [12]. The total volume of each UL was assessed using a measuring tape encircling the analyzed region parallel to the ground. Participants were diagnosed with BCRL according to one of the following criteria: a difference ≥ 200 mL between UL volumes or present a volume ratio > 1.004 (considering the affected UL/unaffected UL) [11].

The truncated cone formula was calculated for all compartments (VC1, VC2, VC3, and VC4), which consists of Example—VC1 = h [P^2^ + (P1 × P2) + P^2^)/12π, where VC is the final volume of the limb segment (VC1—volume of compartment 1 that is between the perimetries (1 and 2); P: perimetry; h: distance between the perimetries (10 cm); π = 3.14159).

### 2.4. Thermographic Images Acquisition

The method for assessing the skin temperature of the UL using thermography followed the four stages recommended by the American Academy of Thermology: acquisition, image processing, delineation of regions of interest (ROI), and analysis. Measurements were conducted in a room devoid of external light and heat-generating electrical equipment, with temperature (23 °C) and relative humidity (55%) controlled by a digital weather station [13].

During scheduling, participants were instructed to adhere to the following precautions: avoid creams or perfumes on the skin, fast for up to three hours, abstain from stimulants, and refrain from vigorous exercise two hours before measurements. Upon arrival, participants were asked to expose the UL, chest, and abdomen for 15 min to achieve thermal equilibrium with room temperature before image acquisition [14].

During image acquisition, participants were positioned standing on a rubber mat in four sequential postures: anterior anatomical, posterior anatomical, and arms abducted in anterior and posterior positions. Saved images were encoded to conceal group allocation to examiners A and B during thermogram analysis.

### 2.5. Camera Model and Calibration

Skin temperature was measured using a portable multispectral thermographic camera ThermoCam FLIR Systems^®^ model C5 (Wilsonville, Oregon, USA) with a range of −20 to 50 °C, infrared spectral band of 8 to 14 µm, and a resolution of 320 × 240 pixels. The camera was set with an emissivity of 0.98 and a focal distance of 1 m.

The device was calibrated with a blackbody source, achieving an uncertainty of less than 0.1 °C (95% confidence) and a stability of ±0.002 °C. Calibration was performed by a certified lab traceable to international standards (RISE and NIST). The blackbody source had an emissivity over 0.995 and an aperture large enough to prevent measurement interference. The camera was placed on a tripod at 75 cm height, with participants positioned 1 m away for accurate image capture of the ROI. Calibration was checked during the acclimatization phase to ensure accurate sensor readings. Studies confirm that this portable camera offers comparable performance to more advanced models for medical diagnostics [8].

### 2.6. Analysis of Thermograms

Thermograms were analyzed using a temperature range from 25 °C to 35 °C using the medical thermal imaging platform (Thermofy^®^, São Paulo, Brazil). A black canvas fixed in the background served as temperature reference. The first four ROI (C1, C2, C3, and C4) were delimited between the perimetry markings previously made (i.e., between P1 and P2, P2 and P3, P3 and P4, and P4 and P5), whereas the last ROI (Cup) corresponded to the polygon encompassing the entire UL region. Independent examiners outlined the polygons for each ROI by encompassing the maximum body segment for analysis and using anatomical contours as a guide to obtain maximum, minimum, and mean temperatures (°C) [15]. Figure 1 illustrates the ROI delineation process.

Each thermographic image underwent three analyses to ensure data reproducibility: two analyses were conducted by the same examiner within a seven-day interval, whereas one analysis was conducted by a second examiner [16]. The examiners, blind to group allocation (with or without BCRL), were experts, trained and calibrated to analyze the thermographic images using the Thermofy^®^ software version 2.1.1.

### 2.7. Data Analysis and Interpretation

The data will be expressed as measures of central tendency. The normality of the data was assessed using the Kolmogorov–Smirnov test. Data were analyzed using SPSS software version 20.0 (IBM Corp, New York, NY, USA) and expressed as mean and standard deviation (SD) or absolute and relative frequency, depending on the nature of the data. For the inter-examiner reproducibility analysis, two independent examiners (A and B), blinded to group allocation, delineated the Regions of Interest (ROI) in each thermogram. Intra-examiner reproducibility was assessed by having examiner A repeat the measurements with a seven-day interval between assessments, following the protocol described by [16]. Both examiners underwent prior training and calibration for thermal image processing using specialized software.

To evaluate intra- and inter-examiner reproducibility, the Intraclass Correlation Coefficient (ICC) was calculated using a two-way mixed-effects model, with single measurements and absolute agreement. The ICC was computed for each ROI in the upper limbs (UL) of women with and without breast cancer-related lymphedema (BCRL). The formula for calculating the ICC is ICC = MS_B_ − MS_W_/MS_B_ + (k − 1) × MS_W_, where MS_B_ is the mean square between subjects (or between examiners), MS_w_ is the mean square within subjects (or within examiners), and k is the number of measurements or raters. This formula quantifies the degree of consistency between the repeated measurements, with ICC values interpreted as follows: Poor: <0.50, Moderate: 0.50 to 0.74, Good: 0.75 to 0.90, and Excellent: >0.90. ICC values provide insight into the reliability of the temperature measurements, indicating the degree of agreement between the two examiners (inter-examiner) and within repeated measurements by the same examiner (intra-examiner).

Data dispersion for the ROIs in ULs with lymphedema was analyzed using the Coefficient of Variation (CV), calculated as CV = (σ/μ) × 100, where σ is the standard deviation and μ is the mean temperature. A CV below 10% indicated low variability and high reproducibility of the measurements, which is crucial for reliable clinical application. Values greater than 10% were considered indicative of high variability in the measurements [16].

To further visualize the distribution of temperature data between Examiner A and Examiner B, histograms were generated for each ROI, showing the maximum, minimum, and mean temperatures. These histograms allowed for a visual comparison of the variability and reproducibility of the temperature readings across different postures.

Finally, a post hoc power analysis was conducted using GPower software (version 3.1) from the University of Düsseldorf. The analysis confirmed that the sample of 30 women (14 with and 16 without BCRL) provided sufficient statistical power (82%) to detect differences in the maximum temperature between the groups. The mean (SD) maximum temperature was 31.53 °C (1.51 °C) for the group with BCRL and 32.90 °C (1.30 °C) for the group without BCRL. Maximum temperature values were used based on recommendations for assessing circulatory changes [17].

## 3. Results

**Figure 2:**
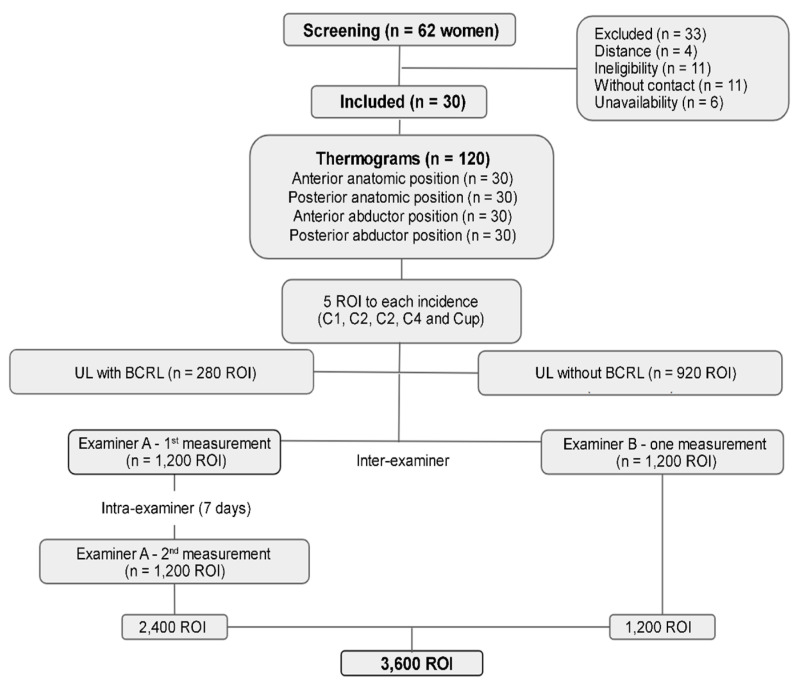
Detailed flowchart of the screening process used for the selection of participants in the thermography study on the upper limbs of breast cancer survivors. The illustration maps the inclusion and exclusion criteria, classifying the upper limbs (UL) as with lymphedema or without lymphedema. Each step of the screening process is represented, from the collection of Regions of Interest (ROI) in four anatomical positions (anterior anatomical, posterior anatomical, anterior abduction, and posterior abduction) to the clinical and thermographic evaluation. The flowchart also demonstrates the intra- and inter-examiner reproducibility analysis, where Examiner A performed two measurements with a 7-day interval, and Examiner B performed one measurement. A total of 3600 ROI were analyzed.

The sample comprised 30 women, 14 with and 16 without BCRL (Table 1). A total of 120 thermograms were acquired and resulted in 1200 ROI per participant: 280 ROI for limbs with lymphedema and 920 for limbs without lymphedema. The analyses by examiners A and B and reanalyses by examiner A resulted in 3600 ROI (Figure 2).

The inter- and intra-examiner reproducibility demonstrated satisfactory ICC indices, ranging from good (0.82 to 0.89) to excellent (0.90 to 1.00) in the limb with and without lymphedema across all regions of interest (ROI), as presented in Table 2 and Table 3. These results highlight the strong agreement between examiners and the reproducibility of thermographic measurements across different postures and ROI.

**Figure 3:**
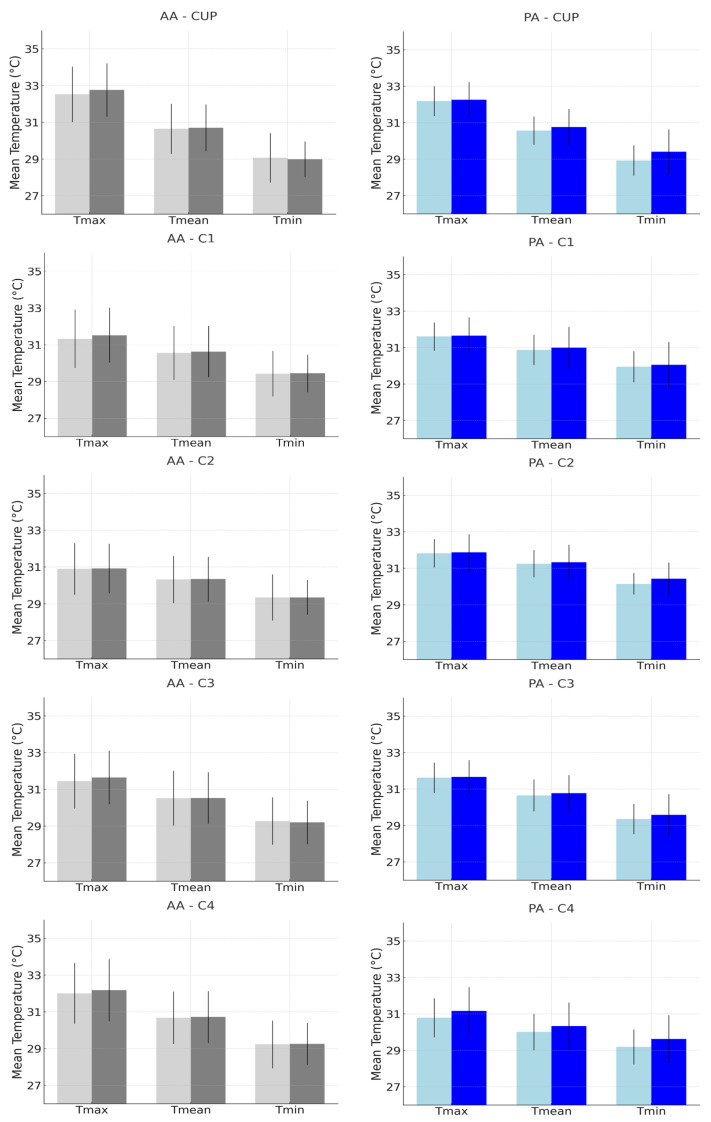
Histograms representing the mean and standard deviation of maximum, mean, and minimum temperatures, comparing Examiner A (lighter bar) with Examiner B (darker bar), for the measurement of regions of interest (CUP, C1, C2, C3, and C4) in the Anatomical Anterior (AA) and Posterior (PA) postures in the upper limb with breast cancer-related lymphedema.

**Figure 4:**
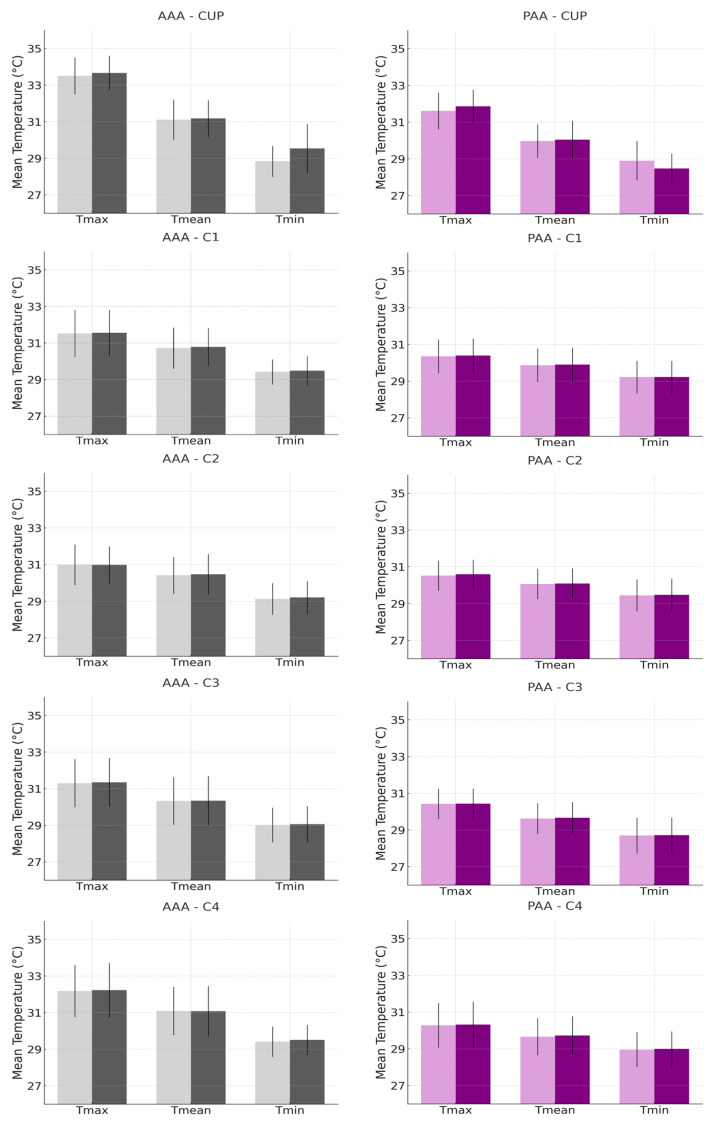
Histograms representing the mean and standard deviation of maximum, mean, and minimum temperatures, comparing Examiner A (lighter bar) with Examiner B (darker bar), for the measurement of regions of interest (CUP, C1, C2, C3, and C4) in the Anterior Arm Abduction (AAA) and Posterior Arm Abduction (PAA) postures in the upper limb with breast cancer-related lymphedema.

Further analysis, detailed in the histograms presented in Figure 3 and Figure 4, reveals the comparison of the mean and standard deviation of the maximum, mean, and minimum temperatures between Examiner A and Examiner B. Figure 3 focuses on the anatomical anterior (AA) and posterior (PA) postures, while Figure 4 examines the anterior and posterior arm abduction postures (AAA and PAA). Minimal differences were observed between the examiners, particularly in the AAA and PAA postures, where variability was lower, reinforcing the reliability of temperature measurements. The coefficient of variation (CV) for the ROI in the upper limb with lymphedema, across all positions, ranged from 1.99% to 5.42%, indicating low variability and strong reproducibility, as detailed in the Supplementary Materials.

## 4. Discussion

The reproducibility of infrared thermography in measuring the skin temperature of the UL of survivors of breast cancer was assessed through an intra- and inter-examiner analysis of thermographic measurements using a protocol that manually delineated the ROI on multispectral images. The thermography showed good-to-excellent reproducibility in the limb with and without lymphedema and low CV values, validating its reliability and reproducibility.

The reproducibility of thermographic measurements was consistent across the four positions assessed, thus reinforcing the reproducibility of the method, offering flexibility in clinical management, and enabling adjustments based on individual needs and specific assessment goals. In this case, the appropriate posture must be selected according to different scenarios (e.g., the need to visualize specific ROI or a precise lymphedema location) and the physical condition of the patient. Positions requiring arm abduction, for instance, may not be suitable for everyone due to the muscular effort needed to support the limb against gravity.

Although certain positions may be more suitable for identifying the areas affected by BCRL, others may offer greater comfort or practicality for the patient and could enhance adherence to the assessment. Moreover, adapting clinical practice to the needs of the patient is important [18]. Therefore, future research should identify other suitable positions based on the clinical and emotional considerations of women with BCRL.

The stability and reproducibility of the results indicate that thermography serves as a reliable test–retest measure capable of assessing the entire UL or specific regions. Considering the heterogeneous distribution of BCRL, which may vary depending on the chronicity, body composition, and severity, thermography may detect changes in skin temperature segmentally [2]. These findings highlight the potential of thermography as a complementary tool for managing BCRL. Also, the ability of thermography to assess the entire limb and specific segments strengthens its reliability by capturing local thermal behavior, thus favoring a more precise therapeutic approach.

Structural and functional changes in BCRL, such as inflammation, lymphatic stasis, adipose tissue proliferation, and fibrosis, may hinder the heat exchange between the affected segment and the environment [19]. Moreover, local metabolic alterations contribute to an imbalance in the autonomic nervous system in maintaining thermoregulation. In this context, thermography is also a valuable tool for detecting metabolic changes and revealing thermal variations suggestive of tissue dysfunction [20,21].

Excellent reproducibility values were observed for all analyzed temperatures. Although mean and maximum temperatures are preferred in clinical practice due to the sensitivity of the minimum temperature relative to the experience level of the examiner that delineates the ROI [22], this study highlights the consistency of the measurement and suggests that the minimum temperature may be a viable option depending on specific objectives.

Unlike more expensive and invasive imaging methods such as lymphoscintigraphy and magnetic resonance imaging (MRI), infrared thermography (IRT) provides real-time images without discomfort or the need for radiopharmaceuticals. This allows the identification of tissue changes in the upper limbs of post-mastectomy women, aiding in the screening, monitoring, and diagnosis of breast cancer-related lymphedema (BCRL), including in its subclinical stage [6]. IRT’s ability to detect early thermal changes increases its potential for early diagnosis and long-term management, contributing to better intervention outcomes and reduced healthcare costs.

In addition, although lymphoscintigraphy is considered the gold standard with moderate-to-excellent reproducibility (ICC 0.70 to 1.00), its use of radioactive tracers and high costs make it less practical for regular monitoring [23]. Bioimpedance spectroscopy, with ICC values between 0.85 and 0.99 [24], is also sensitive to electrode positioning, leading to variability in the results. In contrast, our study showed that IRT demonstrated excellent reproducibility, with ICC values between 0.82 and 1.00, supporting the use of IRT to diagnose breast cancer-related lymphedema [6] and for lymphedema due to filariasis [7]. Given its accessibility and non-invasive nature, IRT is a valuable tool for the continuous monitoring and early detection of BCRL across various care settings, including emergency units, clinics, and private practices.

This study expands the findings of previous research that used objective thermal measurements to assess BCRL [5,6]. In oncology, thermography has gained attention, with evidence supporting its role in monitoring and evaluating the temperature of breast skin in physical exercise programs and therapeutic interventions [14,25]. Moreover, a recent scoping review evidenced infrared thermography as a valuable diagnostic and monitoring tool in healthcare [10].

Skin emissivity was maintained at a constant value to minimize measurement bias [6,26]. Ensuring reliable thermographic measurements involves controlling the ambient temperature and humidity and adhering strictly to variable control measures during thermogram acquisition, including patient preparation and camera adjustments [27].

This study is not free of limitations. Although the relatively small sample size may hinder the generalization of results, the target sample size achieved a sample power of 82%. Longitudinal studies are needed to monitor the thermographic pattern of the BCRL before the clinical application of thermography. Additionally, evaluating the comfort and practicality of the postures is crucial for determining the most appropriate based on BCRL severity and chronicity.

## 5. Conclusions

Infrared thermography (IRT) has proven to be a reliable, practical, and noninvasive imaging tool for assessing skin temperature in the upper limbs (ULs) of breast cancer survivors with and without BCRL. Excellent intra- and inter-rater reproducibility across different regions of interest (ROI) and postures (ICC 0.82–1.0) strongly supports its clinical utility for consistent and repeatable assessments. Although all postures were reproducible for measuring skin temperature, the anterior anatomical position was the most comfortable and is, therefore, the most recommended.

IRT reliably captures the maximum, mean, and minimum temperature measurements, depending on the clinical objective. Furthermore, the coefficient of variation (CV) for all temperature measurements was less than 10%, indicating low inter-rater variability. This demonstrates the robustness of IRT in clinical settings. The ability of IRT to provide non-invasive real-time imaging makes it particularly valuable for the frequent monitoring and early detection of lymphatic dysfunction. Future studies should focus on the longitudinal data to further validate their clinical use, while also exploring the correlation between UL skin temperature and lymphedema stages or severity.

## Figures and Tables

**Table 1:** Sociodemographic and clinical characterization of the sample (n = 30).

Variables		Mean ± SD or n (%)
Age (years)		53.96 ± 8.10
Diagnosis time (months)		94.16 ± 81.18
Time since surgery (months)		85.70 ± 82.67
Body mass index (kg/m^2^)		27.96 ± 4.43
Surgery intervention		
	Modified mastectomy	1 (3.30)
	Radical mastectomy	19 (63.30)
	Simple mastectomy	10 (33.30)
Associated therapies		
	Chemotherapy	29 (96.70)
	Radiotherapy	28 (93.30)
	Hormone therapy	15 (50.00)
Dominance		
	Right-handed	27 (90.00)
	Left-handed	3 (10.00)
Affected UL		
	Right	15 (50.00)
	Left	15 (50.00)
Presence of BCRL		14 (46.70)

UL: upper limb; BCRL: breast cancer-related lymphedema.

**Table 2:** Intra-examiner reproducibility of temperatures measured by thermography.

INTRA-EXAMINER						
	T_max_		T_min_		T_mean_	
Anterior Anatomical Position						
	LWL	LC	LWL	LC	LWL	LC
Cup	1.00 (1.00 to 1.00)	1.00 (0.99 to 1.00)	1.00 (1.00 to 1.00)	0.99 (0.99 to 1.00)	1.00 (1.00 to 1.00)	0.99 (0.99 to 1.00)
C1	0.89 (0.67 to 0.97)	0.99 (0.99 to 1.00)	0.96 (0.89 to 0.98)	0.99 (0.99 to 1.00)	0.89 (0.68 to 0.96)	0.99 (0.99 to 1.00)
C2	0.91 (0.70 to 0.97)	0.99 (0.99 to 0.99)	0.95 (0.84 to 0.98)	1.00 (1.00 to 1.00)	0.91 (0.74 to 0.97)	1.00 (1.00 to 1.00)
C3	0.88 (0.65 to 0.96)	0.99 (0.99 to 0.99)	0.92 (0.77 to 0.97)	1.00 (1.00 to 1.00)	0.95 (0.84 to 0.98)	0.99 (0.99 to 0.99)
C4	0.89 (0.66 to 0.96)	0.99 (0.99 to 0.99)	0.92 (0.75 to 0.97)	1.00 (0.99 to 1.00)	0.92 (0.76 to 0.97)	0.99 (0.99 to 0.99)
**Posterior Anatomical Position**						
Cup	1.00 (1.00 to 1.00)	0.85 (0.73 to 0.92)	0.99 (0.96 to 1.00)	0.86 (0.80 to 0.92)	1.00 (0.99 to 1.00)	0.99 (0.96 to 0.99)
C1	0.99 (0.99 to 0.99)	0.99 (0.99 to 1.00)	0.89 (0.67 to 0.97)	0.99 (0.99 to 1.00)	1.00 (0.99 to 1.00)	0.99 (0.99 to 1.00)
C2	0.99 (0.99 to 0.99)	0.99 (0.99 to 1.00)	0.90 (0.77 to 0.97)	0.99 (0.99 to 1.00)	1.00 (0.99 to 1.00)	0.99 (0.99 to 1.00)
C3	0.99 (0.99 to 1.00)	0.99 (0.99 to 0.99)	0.99 (0.99 to 0.99)	0.99 (0.99 to 1.00)	0.99 (0.99 to 0.99)	0.99 (0.99 to 0.99)
C4	0.99 (0.98 to 0.99)	1.00 (0.99 to 1.00)	0.99 (0.96 to 1.00)	1.00 (0.99 to 1.00)	0.99 (0.99 to 0.99)	1.00 (0.99 to 1.00)
**Anterior Arm Abduction**						
Cup	0.99 (0.99 to 0.99)	0.94 (0.90 to 0.97)	0.94 (0.81 to 0.98)	0.98 (0.96 to 0.99)	0.99 (0.99 to 1.00)	0.95 (0.91 to 0.97)
C1	0.99 (0.98 to 0.99)	0.98 (0.981to 0.99)	0.92 (0.76 to 0.97)	0.82 (0.97 to 0.90)	0.99 (0.97 to 0.99)	0.99 (0.98 to 0.99)
C2	0.99 (0.98 to 0.99)	0.99 (0.99 to 0.99)	0.92 (0.76 to 0.97)	0.97 (0.94 to 0.99)	0.99 (0.98 to 0.99)	0.99 (0.99 to 0.99)
C3	0.99 (0.97 to 0.99)	0.98 (0.97 to 0.99)	0.97 (0.90 to 0.99)	0.93 (0.88 to 0.96)	0.99 (0.97 to 0.99)	0.99 (0.99 to 0.99)
C4	0.98 (0.92 to 0.99)	0.93 (0.89 to 0.96)	0.92 (0.75 to 0.97)	0.92 (0.85 to 0.95)	0.99 (0.96 to 0.99)	0.98 (0.97 to 0.99)
**Posterior Arm Abduction**						
Cup	0.95 (0.92 to 0.97)	0.97 (0.95 to 0.99)	0.92 (0.85 to 0.95)	0.98 (0.94 to 0.98)	0.96 (0.93 to 0.97)	0.98 (0.95 to 0.99)
C1	0.96 (0.92 to 0.98)	0.92 (0.84 to 0.96)	0.96 (0.92 to 0.98)	0.93 (0.89 to 0.97)	0.98 (0.95 to 0.98)	0.92 (0.84 to 0.96)
C2	0.96 (0.91 to 0.98)	0.98 (0.96 to 0.99)	0.95 (0.90 to 0.98)	0.95 (0.89 to 0.97)	0.97 (0.94 to 0.98)	0.98 (0.96 to 0.99)
C3	0.96 (0.90 to 0.98)	0.99 (0.99 to 0.99)	0.91 (0.81 to 0.96)	0.99 (0.98 to 0.99)	0.99 (0.99 to 0.99)	0.99 (0.99 to 0.99)
C4	0.98 (0.94 to 0.98)	0.98 (0.96 to 0.99)	0.95 (0.89 to 0.97)	0.99 (0.97 to 0.99)	0.99 (0.99 to 1.00)	0.99 (0.99 to 1.00)

T_max_—maximum temperature; T_min_—minimum temperature; T_mean_—mean temperature; LWL—upper limb with lymphedema; LC—upper limb without lymphedema. Values represent intraclass correlation coefficients and 95% confidence intervals.

**Table 3:** Inter-examiner reproducibility of temperatures measured by thermography.

	INTER-EXAMINER					
	T_max_		T_min_		T_mean_	
	Anterior Anatomical Position					
	LWL	LC	LWL	LC	LWL	LC
Cup	0.98 (0.94 to 0.99)	0.99 (0.98 to 0.99)	0.95 (0.84 to 0.98)	0.87 (0.77 to 0.93)	0.99 (0.99 to 0.99)	0.99 (0.99 to 0.99)
C1	0.99 (0.99 to 0.99)	0.98 (0.96 to 0.98)	0.90 (0.69 to 0.99)	0.85 (0.72 to 0.91)	0.98 (0.95 to 0.99)	0.98 (0.98 to 0.99)
C2	0.99 (0.97 to 0.99)	0.97 (0.94 to 0.98)	0.95 (0.84 to 0.98)	0.88 (0.78 to 0.93)	0.99 (0.98 to 0.99)	0.98 (0.96 to 0.98)
C3	0.97 (0.92 to 0.99)	0.97 (0.94 to 0.98)	0.95 (0.86 to 0.98)	0.91 (0.84 to 0.95)	0.99 (0.98 to 0.99)	0.98 (0.97 to 0.99)
C4	0.98 (0.95 to 0.99)	0.93 (0.87 to 0.96)	0.94 (0.81 to 0.98)	0.92 (0.86 to 0.95)	0.99 (0.98 to 0.99)	0.96 (0.92 to 0.97)
	**Posterior Anatomical Position**					
Cup	0.97 (0.91 to 0.99)	0.96 (0.92 to 0.98)	0.84 (0.75 to 0.95)	0.88 (0.75 to 0.94)	0.92 (0.76 to 0.97)	0.95 (0.90 to 0.98)
C1	0.99 (0.99 to 0.99)	0.95 (0.90 to 0.98)	0.99 (0.99 to 0.99)	0.90 (0.88 to 0.95)	0.99 (0.98 to 0.99)	0.95 (0.90 to 0.98)
C2	0.99 (0.99 to 0.99)	0.95 (0.89 to 0.97)	0.99 (0.99 to 0.99)	0.90 (0.88 to 0.95)	0.99 (0.98 to 0.99)	0.95 (0.90 to 0.98)
C3	0.93 (0.85 to 0.96)	0.91 (0.83 to 0.96)	0.88 (0.75 to 0.94)	0.92 (0.84 to 0.97)	0.92 (0.82 to 0.96)	0.91 (0.82 to 0.96)
C4	0.87 (0.76 to 0.95)	0.88 (0.75 to 0.94)	0.92 (0.84 to 0.96)	0.93 (0.84 to 0.96)	0.93 (0.85 to 0.96)	0.92 (0.83 to 0.96)
	**Anterior Arm Abduction**					
Cup	0.98 (0.75 to 0.99)	0.99 (0.98 to 0.99)	0.92 (0.89 to 0.99)	0.93(0.86 to 0.96)	0.99 (0.99 to 0.99)	0.99 (0.99 to 0.99)
C1	0.99 (0.99 to 0.99)	0.89 (0.84 to 0.92)	0.99 (0.96 to 0.97)	0.99 (0.98 to 0.99)	0.99 (0.98 to 0.99)	0.98 (0.97 to 0.99)
C2	0.99 (0.98 to 0.99)	0.99 (0.99 to 0.99)	0.99 (0.97 to 0.99)	0.94 (0.88 to 0.97)	0.99 (0.98 to 0.99)	0.99 (0.99 to 0.99)
C3	0.99 (0.97 to 0.99)	0.99 (0.88 to 0.99)	0,95 (0.90 to 0.98)	0.95 (0.89 to 0.97)	0.99 (0.98 to 0.99)	0.90 (0.81 to 0.96)
C4	0.97 (0.94 to 0.99)	0.98 (0.95 to 0.97)	0.95 (0.89 to 0.97)	0.93 (0.84 to 0.96)	0.93 (0.85 to 0.96)	0.92 (0.83 to 0.96)
	**Posterior Anatomical Position**					
Cup	0.94 (0.87 to 0.99)	0.93 (0.85 to 0.96)	0.90 (0.89 to 0.91)	0.88 (0.75 to 0.94)	0.92 (0.84 to 0.96)	0.94 (0.88 to 0.97)
C1	0.93 (0.86 to 0.97)	0.95 (0.89 to 0.97)	0.88 (0.83 to 0.89)	0.89 (0.78 to 0.95)	0.93 (0.86 to 0.97)	0.94 (0.88 to 0.97)
C2	0.93 (0.85 to 0.96)	0,89 (0.77 to 0.95)	0.88 (0.83 to 0.89)	0.80 (0.78 to 0.90)	0.92 (0.84 to 0.96)	0.88 (0.76 to 0.95)
C3	0.86 (0.81 to 0.93)	0,85 (0.79 to 0.93)	0.86 (0.74 to 0.87)	0.88 (0.74 to 0.94)	0.86 (0.80 to 0.93)	0.88 (0.76 to 0.94)
C4	0.93 (0.85 to 0.96)	0.93 (0.86 to 0.97)	0.89 (0.76 to 0.95)	0.90 (0.79 to 0.95)	0.93 (0.85 to 0.97)	0.93 (0.86 to 0.97)

T_max_—maximum temperature; T_min_—minimum temperature; T_mean_—mean temperature; LWL—upper limb with lymphedema; LC—upper limb without lymphedema. Values represent intraclass correlation coefficients and 95% confidence intervals.

## Data Availability

Data supporting the findings of this study are available on request from the corresponding author and are not publicly available due to privacy or ethical restrictions.

## Supplementary Materials

The following supporting information can be downloaded at: https://www.mdpi.com/article/10.3390/biomedicines12112465/s1, Table S1: Coefficient of variation and mean and standard deviation of the temperature analyzed for each region of interest in the upper limb with breast cancer-related lymphedema (n = 14).

## References

[1] Gillespie T.C., Sayegh H.E., Brunelle C.L., Daniell K.M., Taghian A.G. (2018). Breast Cancer-Related Lymphedema: Risk Factors, Precautionary Measures, and Treatments. Gland. Surg..

[2] Brix B., Sery O., Onorato A., Ure C., Roessler A., Goswami N. (2021). Biology of Lymphedema. Biology.

[3] Beelen L.M., Van Dishoeck A.M., Tsangaris E., Coriddi M., Dayan J.H., Pusic A.L., Klassen A., King T. (2021). Patient-Reported Outcome Measures in Lymphedema: A Systematic Review and COSMIN Analysis. Ann. Surg. Oncol..

[4] Matthews M., Gordon S., Witt S., Piller N. (2021). Understanding Normal Seasonal Variations in Upper-Limb Size, Volume and Fluid Distribution in a Healthy Female Population: A North Queensland Study. Wounds Int..

[5] Ibarra Estupiñán A., Pons Playa G., Fernández Garrido M., Zamora Alarcon P., Olivares Dominguez L., Vega García C., Masia Ayala J. (2020). Correlation Between Indocyanine Green Lymphography and Thermography to Evaluate Areas of Dermal Backflow in Lymphedema. J. Plast. Reconstr. Aesthet. Surg..

[6] Dȩbiec-Bąk A., Skrzek A., Wozniewski M., Malicka I. (2020). Using Thermography in the Diagnostics of Lymphedema: Pilot Study. Lymphat. Res. Biol..

[7] Kelly-Hope L.A., Karim M.J., Mahmood A.S., Kawsar A.A., Khair A., Betts H., Douglass J., Forrer A., Taylor M.J. (2020). Infrared Thermal Imaging as a Novel Non-Invasive Point-of-Care Tool to Assess Filarial Lymphoedema. PLoS Negl. Trop. Dis..

[8] Kesztyüs D., Brucher S., Wilson C., Kesztyüs T. (2023). Use of Infrared Thermography in Medical Diagnosis, Screening, and Disease Monitoring: A Scoping Review. Medicina.

[9] (2016). Executive Committee. The Diagnosis and Treatment of Peripheral Lymphedema: 2016 Consensus Document of the International Society of Lymphology. Lymphology.

[10] Borman P. (2018). Lymphedema Diagnosis, Treatment, and Follow-up from the Viewpoint of Physical Medicine and Rehabilitation Specialists. Turk. J. Phys. Med. Rehabil..

[11] Levenhagen K., Davies C., Perdomo M., Ryans K., Gilchrist L. (2017). Diagnosis of Upper Quadrant Lymphedema Secondary to Cancer: Clinical Practice Guideline from the Oncology Section of the American Physical Therapy Association. Phys. Ther..

[12] Campanholi L.L., Baiocchi J.M.T., Batista B.N., Bergmann A., Fregnani J.H.T.G., Duprat Neto J.P. (2021). Agreement Between Optoelectronic Volumetry and Circumferential Girth Measurements to Diagnose Lymphedema in Patients Submitted to Axillary Radical Lymphadenectomy for Treatment of Cutaneous Melanoma. Lymphat. Res. Biol..

[13] American Academy of Thermology (2015). Guidelines for Breast Thermography. Pan Am. J. Med. Thermol..

[14] de Jesus Guirro R.R., Oliveira Lima Leite Vaz M.M., das Neves L.M.S., Dibai-Filho A.V., Carrara H.H.A., de Oliveira Guirro E.C. (2017). Accuracy and Reliability of Infrared Thermography in Assessment of the Breasts of Women Affected by Cancer. J. Med. Syst..

[15] Ammer K. (2008). The Glamorgan Protocol for Recording and Evaluation of Thermal Images of the Human Body. Thermol. Int..

[16] Koo T.K., Li M.Y. (2016). A Guideline of Selecting and Reporting Intraclass Correlation Coefficients for Reliability Research. J. Chiropr. Med..

[17] Formenti D., Ludwig N., Rossi A., Trecroci A., Alberti G., Gargano M., Merla A., Ammer K., Caumo A. (2018). Is the Maximum Value in the Region of Interest a Reliable Indicator of Skin Temperature?. Infrared Phys. Technol..

[18] May-Benson T.A., Teasdale A. (2021). Inter-Rater and Test-Retest Reliability of the Sensory Integration Clinical Observations. Phys. Occup. Ther. Pediatr..

[19] Azhar S.H., Lim H.Y., Tan B.K., Angeli V. (2020). The Unresolved Pathophysiology of Lymphedema. Front. Physiol..

[20] Crandall C.G., Wilson T.E., Kregel K.C. (2010). Mechanisms and Modulators of Temperature Regulation. J. Appl. Physiol..

[21] Romanovsky A.A. (2014). Skin Temperature: Its Role in Thermoregulation. Acta Physiol..

[22] da Silva W., Machado Á.S., Kunzler M.R., Jimenez-Perez I., Gil-Calvo M., Priego-Quesada J.I., Costa P.D., Amorim C.F. (2022). Reproducibility of Skin Temperature Analyses by Novice and Experienced Evaluators Using Infrared Thermography. J. Therm. Biol..

[23] Devoogdt N., Van den Wyngaert T., Bourgeois P., Lambrechts M., Van Kampen M., De Groef A., Geraerts I., Neven P., Vergote I., Tjalma W. (2014). Reproducibility of Lymphoscintigraphic Evaluation of the Upper Limb. Lymphat. Res. Biol..

[24] Ferro A.P., Ferreira V.T.K., Rezende M.S., Souza T.R., de Almeida A.M., Guirro R.R.J., Guirro E.C.O. (2018). Intra- and Inter-Rater Reliability of Bioimpedance in the Evaluation of Lymphedema Secondary to Treatment of Breast Cancer. Lymphat. Res. Biol..

[25] das Virgens Aquino M.J., dos Santos Leite P.M., Lima Rodrigues I.K., DeSantana J.M. (2022). Feasibility for Using Thermography Throughout an Exercise Program in Mastectomized Patients. Front. Oncol..

[26] Oya M., Takahashi T., Tanabe H., Oe M., Murayama R., Yabunaka K., Sanada H. (2016). Low-Temperature Infiltration Identified Using Infrared Thermography in Patients with Subcutaneous Edema Revealed Ultrasonographically: A Case Report. Drug Discov. Ther..

[27] Verstockt J., Verspeek S., Thiessen F., Tjalma W.A., Brochez L., Steenackers G. (2022). Skin Cancer Detection Using Infrared Thermography: Measurement Setup, Procedure and Equipment. Sensors.

