# Supplementary material for: Reproducibility of Thermography for Measuring Skin Temperature of Upper Limbs in Breast Cancer Survivors
Source: Biomedicines. 2024 Oct 27;12(11):2465. doi: 10.3390/biomedicines12112465 (PMC11591836; doi:10.3390/biomedicines12112465)
Supplement: Supplementary file 1 [file biomedicines-12-02465-s001.zip › biomedicines-3253674-supplementary.pdf]

Table S1. Coefficient of variation and mean and standard deviation of the temperature analyzed for each region of interest in the upper limb with breast cancer-related lymphedema (n = 14).

		Tmax				Tmean				Tmin			
**Posture**		**Examiner A**		**Examiner B**		**Examiner A**		**Examiner B**		**Examiner A**		**Examiner B**	
		**Mean (SD)**	**CV %**	**Mean (SD)**	**CV%**	**Mean (SD)**	**CV %**	**Mean (SD)**	**CV %**	**Mean (SD)**	**CV %**	**Mean (SD)**	**CV %**
**AA**	**Cup**	31.53 (1.51)	4.79	31.76 (1.45)	4.57	29.65 (1.36)	4.59	29.70 (1.27)	4.28	28.07 (1.34)	4.77	27.99 (0.97)	3.47
	**C1**	30.33 (1.58)	5.21	30.52 (1.50)	4.91	29.55 (1.46)	4.94	29.63 (1.39)	4.69	28.43 (1.24)	4.36	28.44 (1.02)	3.59
	**C2**	29.90 (1.40)	4.68	29.92 (1.34)	4.48	29.32 (1.27)	4.33	29.34 (1.21)	4.12	28.35 (1.25)	4.41	28.35 (0.94)	3.32
	**C3**	30.45 (1.49)	4.89	30.65 (1.45)	4.73	29.52 (1.49)	5.05	29.53 (1.40)	4.74	28.28 (1.29)	4.56	28.20 (1.18)	4.18
	**C4**	31.009 (1.66)	5.34	31.18 (1.69)	5.42	29.68 (1.43)	4.82	29.72 (1.41)	4.74	28.23 (1.30)	4.61	28.26 (1.13)	4.00
**PA**	**Cup**	31.19 (0.82)	2.63	31.26 (0.98)	3.13	29.57 (0.78)	2.64	29.76 (1.00)	3.53	27.93 (0.83)	2.97	28.41 (1.22)	4.29
	**C1**	30.61 (0.77)	2.52	30.66 (1.00)	3.36	29.88 (0.83)	2.78	30.00 (1.14)	3.80	28.95 (0.85)	2.94	29.06 (1.25)	4.30
	**C2**	30.82 (0.78)	2.53	30.87 (1.00)	3.50	30.25 (0.74)	2.45	30.33 (0.96)	2.83	29.15 (0.58)	1.99	29.42(0.90)	3.06
	**C3**	30.62 (0.84)	2.74	30.67 (0.92)	3.00	29.65 (0.87)	2.93	29.77 (1.00)	3.43	28.36 (0.83)	2.93	28.58 (1.14)	3.99
	**C4**	29.79 (1.07)	3.59	30.16 (1.32)	4.38	29.01 (1.00)	3.52	29.33 (1.30)	4.43	28.19 (0.96)	3.41	28.62 (1.31)	4.58
**AAA**	**Cup**	32.51 (1.00)	3.20	32.67 (0.94)	2.88	30.11 (1.10)	3.65	30.18 (1.00)	3.31	27.84 (0.85)	3.05	28.54 (1.34)	4.70
	**C1**	30.52 (1.28)	4.19	30.56 (1.26)	4.12	29.72 (1.12)	3.77	29.78 (1.04)	3.49	28.42 (0.69)	2.43	28.48 (0.80)	2.81
	**C2**	29.99 (1.11)	3.70	29.98 (1.00)	3.64	29.41 (1.00)	3.50	29.47 (1.10)	3.73	28.13 (0.86)	3.06	28.21 (0.90)	3.19
	**C3**	30.30 (1.32)	4.36	30.35 (1.33)	4.38	29.33 (1.30)	4.43	29.34 (1.35)	4.60	28.01 (0.95)	3.39	28.06 (0.99)	3.53
	**C4**	31.18 (1.43)	4.59	31.23 (1.49)	4.77	30.09 (1.31)	4.35	30.08 (1.38)	4.59	28.42 (0.83)	2.92	28.51 (0.82)	2.88
**PAA**	**Cup**	30.61 (1.00)	3.33	30.86 (0.90)	2.92	28.97 (0.92)	3.18	29.05 (1.02)	3.51	27.90 (1.07)	3.84	27.48 (0.82)	2.98
	**C1**	29.35 (0.92)	3.13	29.39 (0.93)	3.16	28.87 (0.91)	3.15	28.90 (0.91)	3.15	28.22 (0.89)	3.15	28.22 (0.89)	3.15
	**C2**	29.52 (0.81)	2.74	29.59 (0.79)	2.67	29.07 (0.82)	2.82	29.10 (0.82)	2.82	28.45 (0.87)	3.06	28.48 (0.88)	3.09
	**C3**	29.42 (0.83)	2.82	29.43 (0.83)	2.82	28.62 (0.85)	2.97	28.67 (0.85)	2.96	27.70 (0.97)	3.50	27.72 (0.97)	3.50
	**C4**	29.28 (1.21)	4.13	29.32 (1.25)	4.26	28.67 (1.005)	3.66	28.73 (1.04)	3.62	27.96 (0.95)	3.40	28.00 (0.95)	3.39

Tmax: maximum temperature; **Tmin:** minimum temperature; **Tmean**: mean temperature; **SD**: standard deviation; **CV**: coefficient of variation; **AA**: anterior anatomical; **PA**: posterior anatomical; **AAA**: anterior arm abduction; **PAA:** posterior arm abduction.
